# Unilateral deposition of paramagnetic substances in basal ganglia as early sign of neurodegeneration in magnetic resonance imaging

**DOI:** 10.3389/fneur.2026.1917665

**Published:** 2026-10-26

**Authors:** Ibone Saralegui Prieto, Jose Mari Ontañón Garcés, Begoña Fernandez-Ruanova, Jofre Tenorio-Laranga, Iñigo Vicente Olabarria

**Affiliations:** 1 Osakidetza, Osatek, Unit of Galdakao, Galdakao, Biscay, Spain; 2 Osakidetza, Osatek, RDI Department, Bilbao, Biskay, Spain; 3 Biobizkaia Health Research Institute, Barakaldo, Spain

**Keywords:** basal ganglia, iron deposition, magnetic resonance, susceptibility weighted imaging sequence, transynaptic degeneration

## Abstract

**Purpose:**

To describe a new radiological finding—an asymmetric deposition of paramagnetic substances in the ipsilateral basal ganglia to a mainly frontal supratentorial lesion—retrospectively observed via Magnetic Resonance (MR), using a Susceptibility Weighted Imaging sequence (SWI), and develop a reliable hypothesis of its possible origin in the context of a transsynaptic degeneration.

**Methods:**

Following initial clinical observations of this specific MR sign, a systematic retrospective database review of the more recent reports of any case with a history of tumors or other frontal pathology was conducted, to evaluate using the SWI sequence if this asymmetric deposition of paramagnetic substances in the basal ganglia was consistently present. In addition, the identified cases (frontal pathology with asymmetric deposition of paramagnetic substances in the basal ganglia) showed signs of a pathology different from the one mentioned above, or in other areas of the brain, that could justify the presence of these deposits.

**Results:**

Within the retrospectively evaluated cohort of 35 patients, the primary supratentorial etiologies were diverse, from tumors, ischemic pathology or previous surgery, and the predominant location was the frontal lobes. Different symptoms were present in 54.3% of the patients. According to the qualitative analysis carried out using the SWI sequences, this deposition tended to occur predominantly in the globus pallidus and substantia nigra ipsilateral to a supratentorial lesion. Furthermore, 11 out of the 35 cases (32%) had incipient anterograde degeneration of the ipsilateral corticospinal pathway. It should also be noted in post-surgical patients that this asymmetric deposition of paramagnetic substances did not exist in the studies prior to the intervention.

**Conclusion:**

It is hypothesized that this structural asymmetry represents an early sign in MR in the context of transsynaptic degeneration with associated iron deposition in the basal ganglia. This hitherto undescribed finding could be important to further knowledge of the pathophysiology of basal ganglia, and could serve as a biomarker and prognostic factor in patients with frontal cortex pathology.

## 1 Introduction

Both the cytoarchitecture of the basal ganglia and their connectivity with the rest of the structures of the brain are complex, and the mechanisms of degeneration and their impact on the functioning of the rest of the brain are still the reason for numerous researches.

In this section, an anatomical and functional overview of the basal ganglia is provided, alongside a review of physiological and pathological paramagnetic deposits and secondary neurodegenerative mechanisms. This synthesis establishes the rationale for the hypothesis of a potential transsynaptic degeneration with associated iron deposition in the basal ganglia secondary to a frontal ipsilateral noxa, based on an asymmetrical deposition of paramagnetic substances in the basal ganglia visualized on MRI.

### 1.1 Structural organization and connectivity

The basal ganglia, in close coordination with the cerebellum exert a critical regulatory influence over motor activity initiated within the cerebral cortex. These subcortical structures modulate the planning, initiation, and execution of voluntary movements through complex, re-entrant feedback loops involving the thalamus. By processing cortical inputs and returning refined signals, the basal ganglia ensure the precision, speed, and postural stability of directed actions. This regulatory mechanism effectively filters cortical output via the pyramidal pathway, selectively reinforcing desired motor outcomes while suppressing deleterious or competing signals (1, 2).

Beyond pure motor This integrative capacity extends into the cognitive and emotional spheres; the basal ganglia are essential mediators in higher-order processes, including executive functions such as working memory and decision-making, as well as the dopaminergic modulation of reward, motivation, and affective processing (3).

Architecturally, the basal ganglia comprise a complex of subcortical nuclei embedded within the deep cerebral white matter.

The neostriatum (comprising the caudate nucleus and the putamen) functions as the principal input unit, integrating excitatory glutamatergic signals from the cerebral cortex (1).

The ventral striatum—encompassing the nucleus accumbens and the olfactory tubercle—are involved in the “reward circuit,” fundamentally integrated into the limbic system.

The paleostriatum (globus pallidus), divided into internal (GPi) and external (GPe) segments, alongside the subthalamic nucleus (STN) and the substantia nigra (SN), process and refine these signals. The SN is partitioned into the pars compacta (SNpc), critical for nigrostriatal dopaminergic signaling, and the pars reticulata (SNpr). The SNpr serves as a major efferent station, characterized by integrative synaptic capabilities that transmit inhibitory signals back to the thalamus (4).

The functional architecture of the basal ganglia is defined by an organization of multiple parallel loops and re-entrant circuits (5). Each of these circuits eventually converges on distinct territories within the frontal lobe, including the pre-central motor fields, the frontal eye fields, and the orbitofrontal cortex (3, 5). Basal ganglia regulate motor function through various pathways to initiate, end, or regulate the extension of movements. These pathways are: Direct (excitatory, Pro-kinetic; Figure 1), Indirect (inhibitory, Anti-kinetic; Figure 2), and Hyperdirect (Global Inhibition; Figure 3), depending on whether the striatum projections are directed to the GPe or the GPi (1, 6).

**Figure 1:**
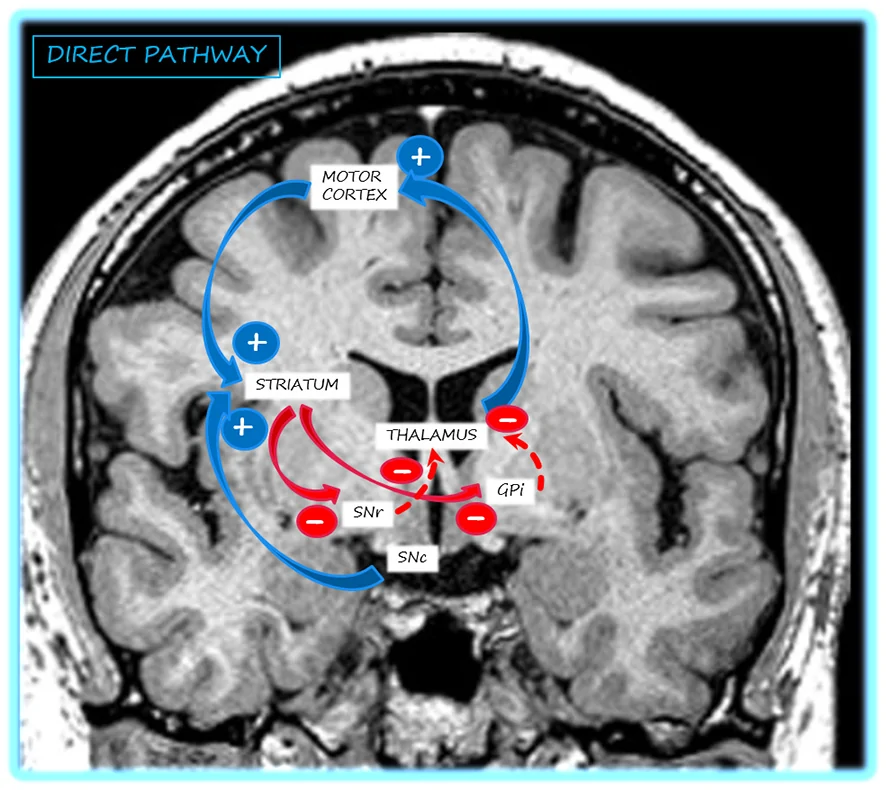
Direct Pathway. Triggered by excitatory cortical input, activating the inhibitory neurons of the striatum. The striatal neurons project toward the internal segment of the globus pallidus (GPi). GPi neurons are inhibitory, releasing GABA onto the thalamus. Inhibited GPi sends decreased inhibitory projections to the thalamus, disinhibiting the thalamus. The thalamus then sends a greater excitatory output to the cortex.

**Figure 2:**
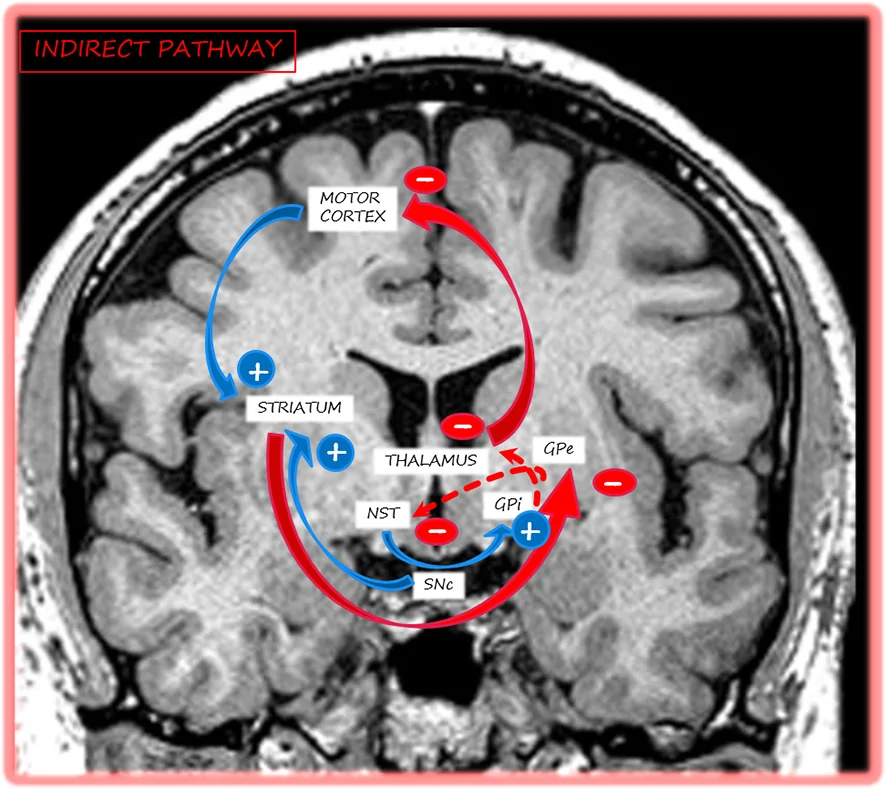
Indirect Pathway. Cortical neurons excite striatum inhibitory neurons, which inhibit neurons on the external segment of the globus pallidus (GPe), which sends inhibitory fibers to the subthalamic nucleus (STN). The subthalamic nucleus sends its axons to the internal segment of the globus pallidus (GPi) and to the reticular portion of the substantia nigra (SNpr), and from there they continue as the direct pathway with GABAergic inhibitory neurons to the thalamus. Functionally, the neostriatum inhibits the external globus pallidus, causing disinhibition of the subthalamus.

**Figure 3:**
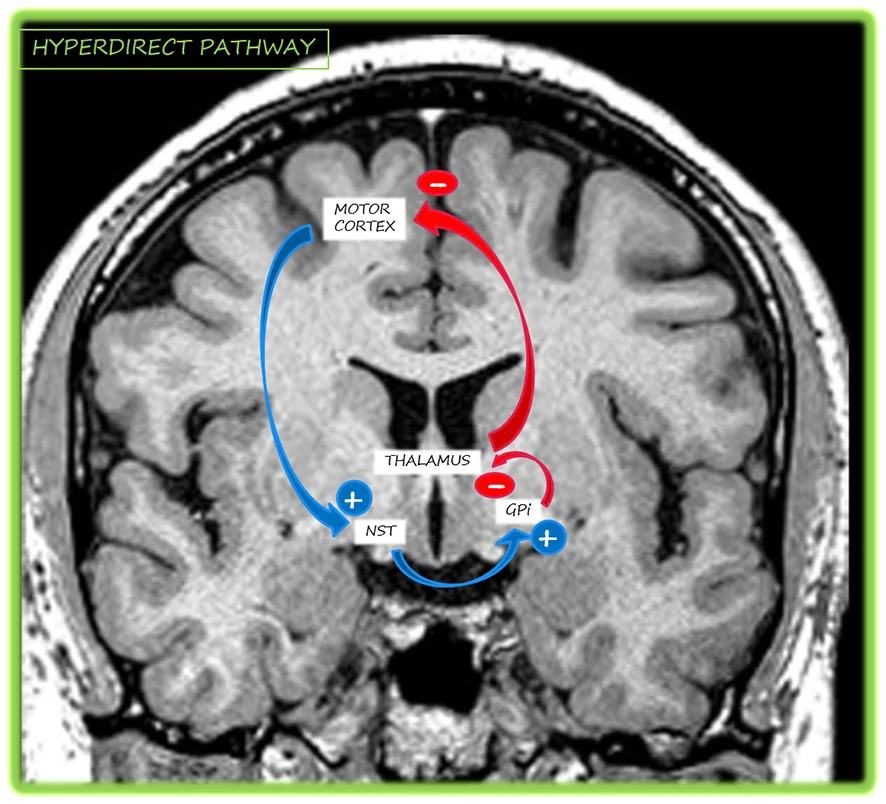
Hyperdirect pathway. The cortex sends excitatory signals directly through the subthalamic nuclei (STN) to the internal segment of the globus pallidus (GPi) and the reticular portion of the substantia nigra (SNpr), bypassing the neostriatum. The glutamatergic excitatory neurons in NSTs can excite the GPi/SNpr, GPi/SNr, suppressing thalamic activity in the cerebral cortex and increasing inhibitory influences on upper motor neurons.

Consequently, structural disruption of frontal cortical areas directly impacts the structural and metabolic integrity of these highly topographically organized downstream subcortical targets.

### 1.2 Age-related physiological iron deposition in the basal ganglia and cerebral cortex

Various physiological changes over a lifetime, including volume loss, myelin degeneration, and regional iron accumulation, all of which can be monitored *in vivo* using advanced magnetic resonance imaging (MRI) techniques. Distinguishing these age-related changes from true pathological processes is essential for neurobiological evaluation.

The magnetic susceptibility of brain tissue is mainly determined by the ferritin-iron content in the basal ganglia and other gray matter structures (7) and may be influenced by myelin, calcium, or deoxyhemoglobin. Physiological iron accumulation occurs heterogeneously, demonstrating a strong predilection for regions involved in motor and cognitive functions, most notably the globus pallidus, putamen, substantia nigra, and red nuclei (8).

Structures accumulating iron with age frequently contain iron-dependent dopaminergic pathways, and it has been hypothesized that physiological variations in iron content and dopaminergic transmission may modulate behavioral changes during normal aging (9, 10). While physiological iron accumulation follows a predictable, highly symmetrical linear or quadratic pattern during adult life in healthy individuals, abnormal regional variations—such as interhemispheric asymmetry—might indicate an underlying focal pathological process rather than normal aging.

### 1.3 Pathological iron homeostasis and neurodegeneration

Iron is involved in numerous functions in the brain, such as oxygen transport, oxidative phosphorylation, DNA synthesis, mitochondrial respiration, myelin synthesis, activation of antioxidant enzymes, and neurotransmitter metabolism (8). However, the alteration of iron homeostasis can trigger significant neurotoxicity. Iron accumulation within neurons can induce damage via apoptotic pathways, while glial iron overload triggers a pro-inflammatory state characterized by the release of pro-inflammatory cytokines, initiating a self-propelling cycle of neuroinflammation and tissue degradation (11). These two mechanisms mutually enhance each other, creating a deleterious feedback loop that exacerbates neuronal death.

Neurodegenerative diseases with iron deposition are classified as acquired and genetic neurological disorders. Inherited diseases, collectively referred to as Neurodegeneration with Brain Iron Accumulation diseases (NBIA). Conversely, acquired neurodegenerative conditions—including Alzheimer's disease, Parkinson's disease, amyotrophic lateral sclerosis, multiple sclerosis, progressive supranuclear palsy, and corticobasal degeneration—frequently display secondary iron overload within the SNpc (12). Throughout the aging process, the basal ganglia exhibit an increased vulnerability to iron accumulation, exposing these structures to an elevated risk of neuroinflammation. Connected through intricate molecular interactions, local inflammation and paramagnetic iron deposition establish a harmful environment that sustains progressive structural degeneration.

### 1.4 Secondary neurodegeneration and transsynaptic dynamics

Neurodegeneration represents a progressive process culminating in neuronal death and functional compromise of the nervous system.

Distal or secondary neuronal degeneration defines the process of demyelination and disintegration of a neuron or neuronal circuit as a consequence of a distant localized injury.

Secondary neurodegeneration is broadly categorized into axonal degeneration and transneuronal (or transsynaptic) degeneration (13).

Axonal degeneration refers to the loss of structural integrity and function of axons following an injury to the neuronal soma or the proximal axon, progressing either in an anterograde or retrograde fashion without crossing synaptic boundaries (13).

In contrast, transsynaptic degeneration involves structurally intact neurons that are synaptically connected to the primary damaged cells. This phenomenon represents afferent or efferent neuronal degradation propagating through synaptic junctions due to the absolute loss of synaptic input or output (14). Transsynaptic degeneration can also be anterograde and retrograde.

The functional concept of transsynaptic disruption traces back to the term “diaschisis,” coined by Von Monakow to describe the cessation of function in remote brain regions anatomically connected to a primary site of injury. While early definitions of diaschisis excluded macro-structural adjustments, recent histopathological and radiological studies have confirmed that secondary degeneration causes tangible morphological modifications, tissue necrosis, and gliosis in remote regions. For instance, following a cerebral infarction within the middle cerebral artery (MCA) territory, neuronal death and gliosis can be detected in the ipsilateral thalamus and the substantia nigra, both situated far outside the primary vascular territory (15).

This secondary neurodegenerative process occurs selectively along connected pathways weeks, months, or years after the primary insult, and its inflammatory response represents an early event driven by cytokine upregulation and microglial/astrocytic activation (16). Because active microglia and reactive astrocytes act as local scavenger cells that accumulate metabolic residual iron during the phagocytosis of degenerated tracts, Susceptibility-Weighted Imaging (SWI) serves as a highly sensitive tool to capture these localized, microenvironmental paramagnetic shifts. Recent studies suggest that secondary degeneration is associated with neurological deficits and may predict motor outcome after supratentorial injury.

To date, a significant clinical and radiological gap remains regarding the *in vivo* tracking of transsynaptic degeneration within frontostriatal pathways. To address this question, a series of retrospective clinical cases is presented as preliminary observations supporting the hypothesis that primary supratentorial frontostriatal disruption leads to a persistent asymmetric deposition of paramagnetic substances in the ipsilateral basal ganglia, thereby offering a potential surrogate imaging marker for transsynaptic neurodegeneration.

## 2 Material and methods

### 2.1 Study design and case selection

Following initial serendipitous observations of an asymmetric deposition of paramagnetic substances in the basal ganglia in MR, a retrospective, exploratory case-series was designed. The primary objective was to determine whether this radiological finding recurred systematically in patients with specific supratentorial lesions, thereby establishing a preliminary framework for its underlying pathophysiological mechanism.

A retrospective review of the institutional database at Galdakao Hospital (Bizkaia) during 2023 and 2024 was performed. A total of 35 patients demonstrating a distinct deposition of paramagnetic substances in the basal ganglia were included. Given the exploratory nature of this study, inclusion was based on the presence of this specific radiological asymmetry to systematically characterize its syndromic and structural associations.

### 2.2 MRI acquisition protocols

All the MRI had been performed using either 3T (Achieva, Phillips SA) or 1.5T (Aera or Sola, Siemens) scanners equipped with standard multi-channel head coils. The imaging protocols included standard morphological sequences: 2D or 3D sequences, sagittal T1-weighted, T2-weighted, and Fluid-Attenuated Inversion Recovery (FLAIR) sequences, alongside axial diffusion-weighted imaging (DWI) and 3D susceptibility-weighted imaging (3D-SWI).

The acquisition parameters for the 3D-SWI sequences were standardized as follows:

- **3T Protocol:** TR/TE1/delta TE: 31/7,2/6,2 ms; phase direction: R-L; FoV: 230 x 150 x 130 mm; matrix: 384 x 264; resolution: 0.6 x 0.6 x 2; **–**0,1 gap; 140 slices. *t: 2 min*.
- **1.5T Protocol:** TR: 49 ms; TE: 40 ms; angle: 15; phase direction: R-L; FoV: 245 mm; matrix: 320 x 320; resolution: 0.4 x 0.4 x 2.3; 20% gap; 60 slices. *t: 2,56 min*.

Each 3D-SWI sequence included: magnitude, phase, minimum intensity projection (MIP), and fully processed SWI datasets were automatically online reconstructed. All datasets were independently evaluated by two experienced neuroradiologists, with consensus reached in all cases.

### 2.3 Image analysis and differential diagnosis

Morphological sequences (T1, T2, and FLAIR) were systematically reviewed to evaluate primary supratentorial pathologies (e.g., ischemic lesions, tumors, gliosis, tissue necrosis, or demyelination). In these conventional sequences, the baseline anatomical appearance of the deep gray matter nuclei was confirmed to be symmetrical and devoid of macroscopic parenchymal distortion or signal abnormalities that could suggest independent primary basal ganglia disease.

A qualitative and comparative analysis of the SWI datasets was performed. The use of different magnetic field strengths (1.5T and 3T) did not influence the qualitative assessment of regional asymmetry. Magnitude images were utilized to visually confirm the interhemispheric asymmetry of the paramagnetic substance deposition within the subcortical nuclei.

Strict radiological exclusion criteria were applied using multi-sequence correlations to rule out alternative confounding causes of SWI hypointensity:

- **Exclusion of calcifications:** mineralization was ruled out utilizing filtered SWI phase images, ensuring that the target hypointensities demonstrated a true paramagnetic shift (inverse to the diamagnetic signal intensity characteristic of calcium).
- **Exclusion of blood products and vascular anomalies:** subacute or chronic hemorrhagic remnants (such as intracellular methemoglobin or focal hemosiderin sequelae from local microbleeds) and prominent vascular structures (such as medullary or engorged veins) were excluded based on the absence of corresponding signal alterations on conventional T1, T2, and FLAIR sequences, as well as the absence of tubular or anomalous vascular morphology within the involved nuclei.

### 2.4 Characterization and evolution

Following definitive cohort selection, the specific involvement of individual deep nuclei and their potential structural extension to adjacent brain regions were documented. For patients with available longitudinal follow-up or prior imaging datasets, the temporal development and evolution of the paramagnetic deposit were analyzed in relation to the timeline of the primary supratentorial injury.

Due to the retrospective design of this historical imaging cohort, raw phase data suitable for *post-hoc* quantitative susceptibility mapping (QSM) reconstruction were unavailable. Consequently, no absolute quantification of magnetic susceptibility was performed.

## 3 Results

### 3.1 Demographic and clinical characteristics of the cohort

The retrospective analysis of the 35 brain MRI datasets included 13 female (37.1%) and 22 male (62.9%) patients. The ages ranged from 15 to 84 years, with a calculated mean age of 61.4 years.

The primary clinical etiologies distributed across the cohort were categorized as follows: 11 cases of established cerebral infarction (Figure 4), 11 patients who underwent surgical resection for a brain tumor, 7 patients with subcortical leukoencephalopathy (including 4 with ischemic microangiopathy, 1 with neuro-Behçet's disease, 1 with post-traumatic encephalomalacia, and 1 with a bilateral frontoparietal manifestation secondary to Wilson's disease), 2 cavernous hemangiomas, 1 low-grade glial tumor localized to the thalamus (Figure 5), 1 supratentorial cerebral hematoma, 1 extensive supra- and infratentorial superficial siderosis, and 1 patient diagnosed with multiple sclerosis (MS) presenting an active chronic periventricular or smoldering lesion.

**Figure 4:**
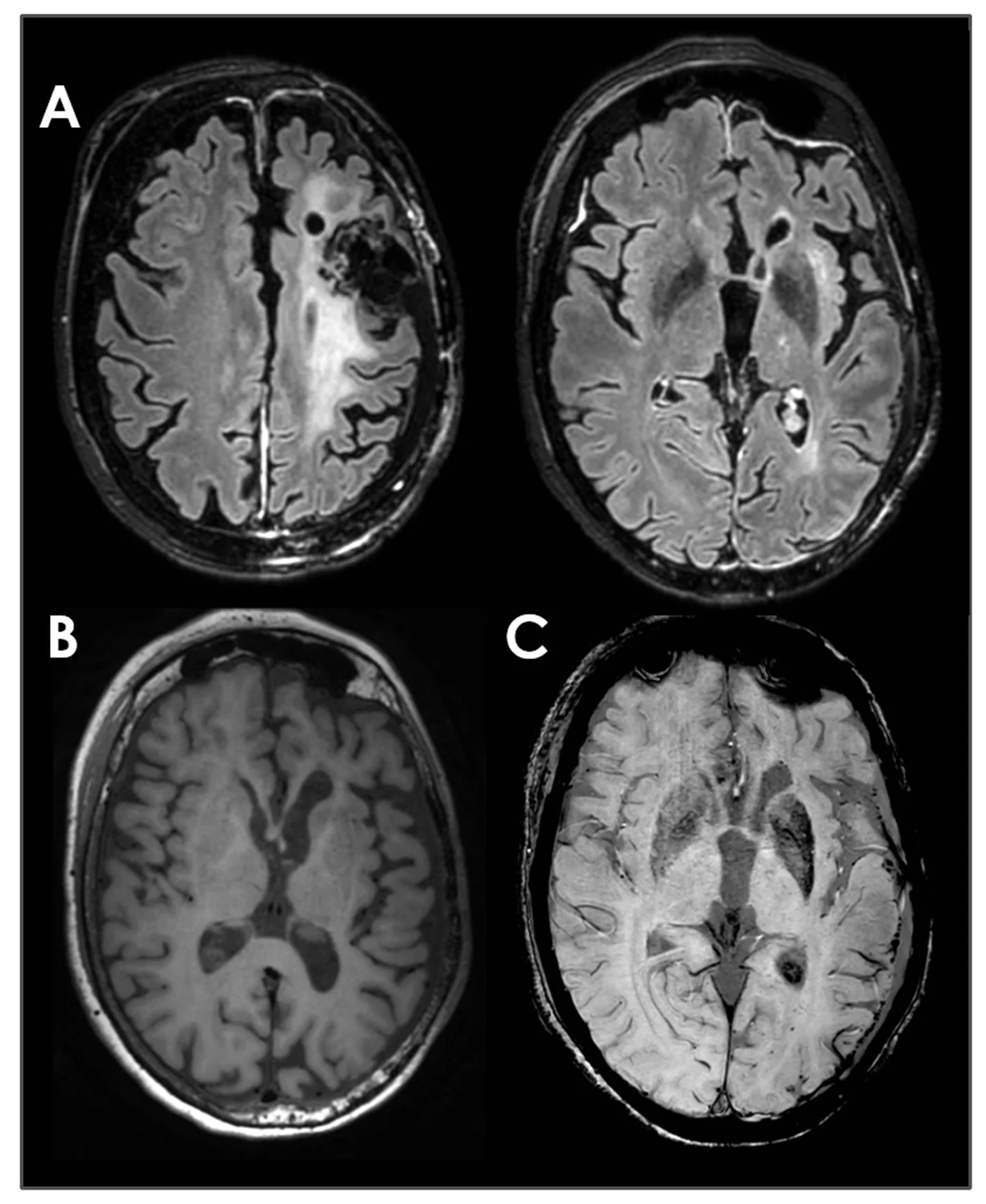
FLAIR **(A)**, T1WI **(B)** and SWI **(C)** sequences. Postoperative encephalomalacia in the left middle and lower frontal gyri, with subcortical leukopathy in this cerebral hemisphere. Associated asymmetric deposition of paramagnetic substances in the left lenticular nucleus **(C)**.

**Figure 5:**
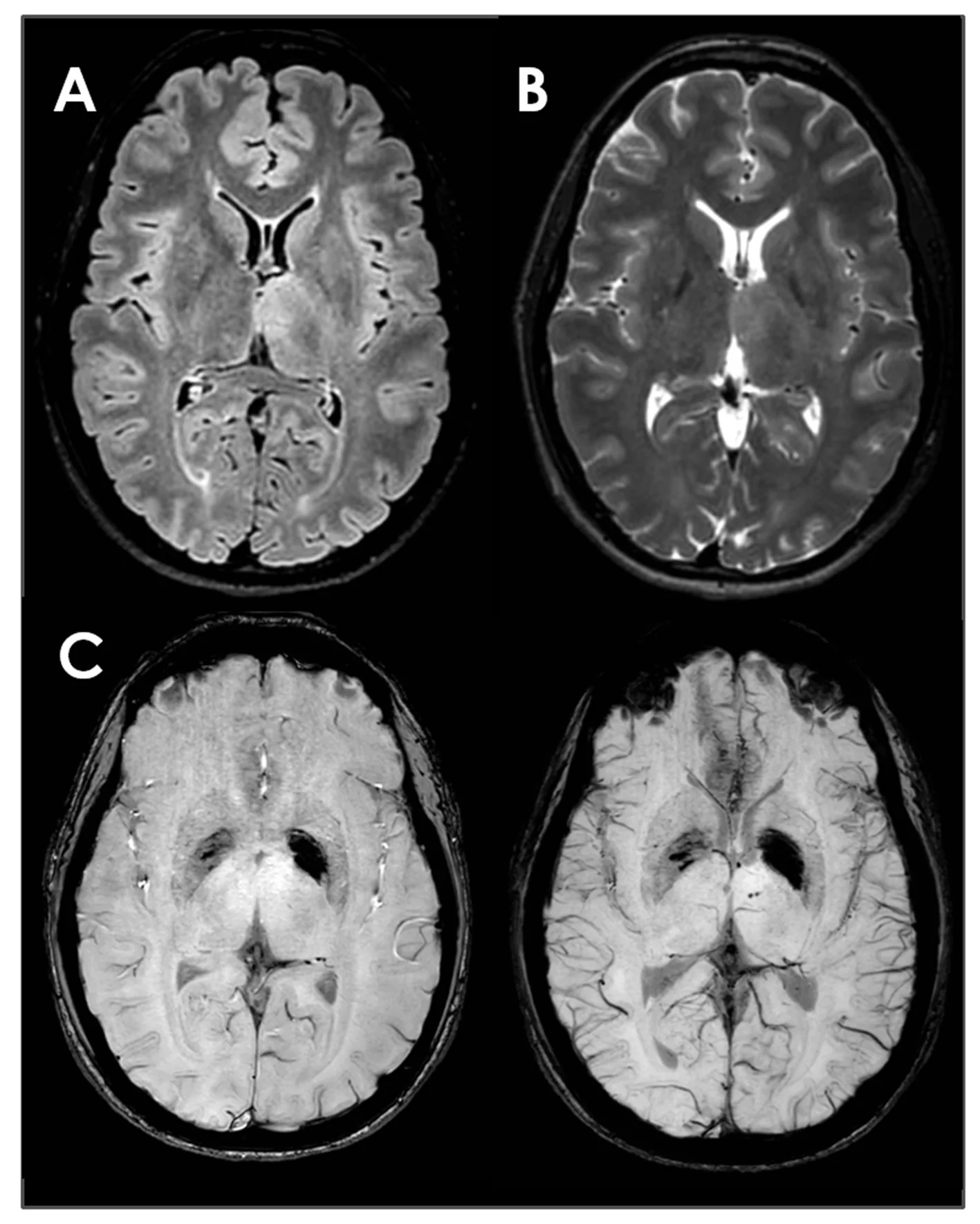
FLAIR **(A)**, T2WI **(B)** and SWI **(C)** sequences. Left thalamic tumor, with associated asymmetric deposition of paramagnetic substances in the left globus pallidus **(C)**.

Based on the medical history at the time of evaluation, sixteen of those 35 patients did not have significant neurological symptoms. In contrast, the remaining 19 symptomatic patients demonstrated heterogeneous clinical manifestations: five patients had instability, three dysmetria and dystonia, three paresthesias, three hemiplegia, one bradykinesia, one tremor with hyperreflexia, one intermittent claudication, one facial paresis and the patient with Wilson's disease presented a complex and severe clinical manifestation. It should be noted, however, that in this retrospective study, all the examinations were follow-up MRI, and the vast majority of symptomatic patients displayed deficits predominantly related to their primary underlying neurological pathology.

### 3.2 Topographical distribution of primary lesions

Anatomical mapping of the primary structural injuries demonstrated a distinct predilection for the frontal lobes, accounting for 19 cases (9 located within the right frontal lobe and 10 within the left frontal lobe). There were also two right parietal lesions; three left temporal and one left occipital; one left thalamic tumor (Figure 5). Regarding deep gray matter boundaries, primary lesions were identified in the left striatum (*n* = 2) and the right striatum (*n* = 3), alongside one lesion involving the ventricular system. Extensive structural damage was documented in three patients, corresponding to the cases of: one diffuse bilateral leukoencephalopathy, bilateral frontoparietal Wilson's disease, and 1 extensive superficial siderosis.

### 3.3 SWI sequence

The most important findings in the SWI sequence were: a unilateral asymmetric deposition of paramagnetic substances was observed in this sequence, which did not correspond to calcium in the phase images, predominantly in the globus pallidus and SN ipsilateral to a supratentorial lesion, in 16 out of the 35 cases (46%). In six cases, the asymmetric deposition of these paramagnetic substances occurred in the striatum; in five in this striatum and the SN; in three only in the globus pallidus; and in one case in the lenticular nucleus and the SN.

In two cases, this abnormal deposition of paramagnetic substances occurred bilaterally, in a patient with Wilson's disease, and in the case of diffuse superficial siderosis. These two cases support our hypothesis that transsynaptic degeneration in the basal ganglia is associated with a deposition of paramagnetic substances.

In two other cases, there was no significant asymmetric deposition of paramagnetic substances in the basal ganglia in relation to the contralateral side, in a patient with an operated anterior temporal tumor, and in a patient with a previous infarction in the striatum. In these two cases, the asymmetrical deposit was not significant, which may be related to the location of the lesion, which, based on our collected data, seems to have a higher prevalence when the lesion is located at the frontal level, that is, in the motor circuit.

### 3.4 Subgroup analysis according to primary etiology and topographical distribution

The patterns of asymmetric paramagnetic substances deposition across the basal ganglia were stratified based on the primary underlying supratentorial pathologies.

- In the established infarction subgroup (*n* = 11), asymmetric deposition was confirmed in 10 cases. The regional distribution was predominantly localized in the ipsilateral globus pallidus and SN in five cases, confined to the striatum in three cases, and involved both the striatum and the SN in two cases.
- From the patients undergoing surgical resection for brain tumors (*n* = 11), the asymmetric deposition was mapped in the ipsilateral globus pallidus and SN in six cases, in the striatum in two cases, in the striatum and SN in one case, and restricted to the globus pallidus in one case. In one patient within this group, the interhemispheric asymmetry did not reach radiological significance.
- Within the subcortical leukoencephalopathy cohort (*n* = 7), the asymmetric deposit was identified in the ipsilateral globus pallidus and SN in three cases, in the striatum and SN in one case, and within the striatum in one case. The case of post-traumatic encephalomalacia, this deposition occurred in the ipsilateral lenticular nucleus and the SN, and in the patient with bilateral frontoparietal involvement due to Wilson's disease this deposition occurred bilaterally.
- In the remaining low-prevalence etiologies, both patients presenting with cavernous hemangiomas demonstrated this asymmetrical deposition within the ipsilateral lenticular nucleus and the SN. The patient diagnosed with a thalamic low-grade glial tumor exhibited isolated deposition in the ipsilateral globus pallidus. The patient with Superficial Siderosis also had a global bilateral involvement of the basal ganglia. Finally, the case of a right parietal cerebral hematoma showed this deposition within the right striatum and the SN, while the patient with MS showed this deposition in the left globus pallidus.

### 3.5 Evolution over time of this asymmetric deposition of paramagnetic substances

Longitudinal imaging data provided key insights into the chronological development of this asymmetrical deposition relative to the primary brain injury.

- In the ischemic stroke subgroup (*n* = 11), baseline MRI datasets prior to the acute vascular event were unavailable. However, serial follow-up scans in two patients demonstrated a progressive, slight increase in the regional paramagnetic deposit, at 1-year (1); and at 2-year, concurrently with signs of secondary Wallerian degeneration (1).
- From the 11 patients operated on for a brain tumor, eight had pre-surgical MRI, all without abnormalities in the basal ganglia in the SWI sequence.

In seven of these patients the deposit took between 1–3 years to produce, and only this deposit increased slightly in three patients.

- In relation to the seven patients with a leukopathy, five patients had follow-up MRI, where a latency period between 2 and 5 years was observed to manifest that asymmetrical deposition, either by a measurable objective increase in the underlying lesion load (*n* = 2) or by the structural evolution of lesions situated in strategic frontostriatal pathways (*n* = 3). In contrast, the patient with Wilson's disease demonstrated severe bilateral deep gray matter involvement on the initial baseline MRI, which remained structurally stable during successive controls.

### 3.6 Corticospinal pathway involvement and clinical manifestations

Concomitant incipient anterograde degeneration of the ipsilateral corticospinal tract was radiologically identified in 11 out of the 35 cases (32%), four patients with established infarction, three with subcortical leukoencephalopathy, three patients with operated on for a brain tumor, and one patient with a supratentorial haematoma (Figure 6).

**Figure 6:**
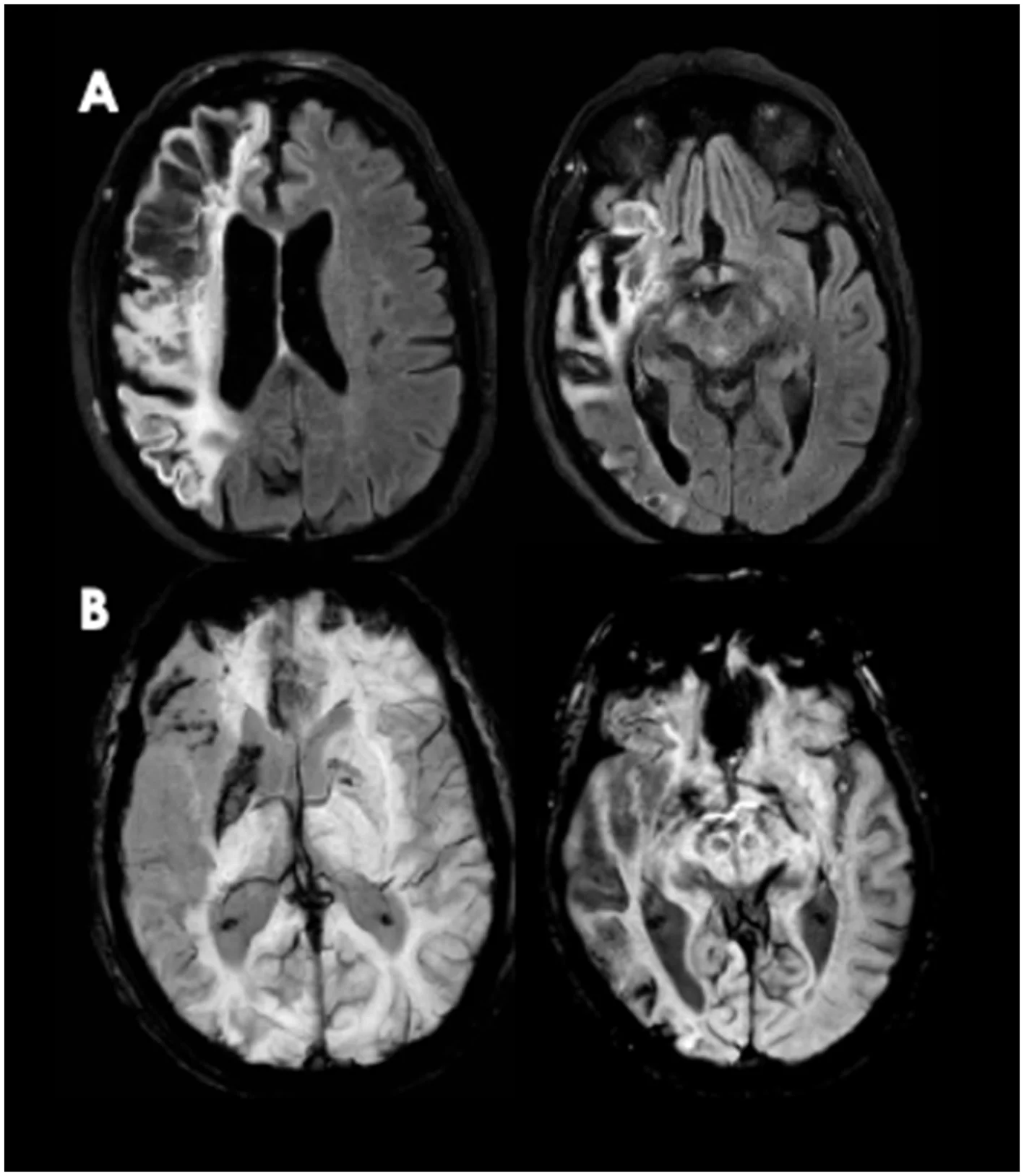
FLAIR **(A)** and SWI **(B)** sequences. Extensive area of right frontoparietal postischemic encephalomalacia, with subcortical leukopathy and expansion of this ventricle, and incipient wallerian degeneration, with mild gliosis in the right cerebral peduncle **(A)**. Asymmetric deposition of paramagnetic substances in the striatum and ipsilateral SN **(B)**.

Clinically, three of these 11 patients remained completely asymptomatic during routine follow-up. The remaining eight patients exhibited heterogeneous neurological manifestations including, one dystonia, one bradykinesia, one intermittent claudication, one gait instability, three hemiplegia and one altered facial sensitivity.

In summary, an asymmetric deposition of paramagnetic substances was identified within the basal ganglia, secondary to an ipsilateral, predominantly frontal supratentorial injury (Figure 7). Notably, this accumulation was not associated with other local morphological changes in the basal ganglia in the other morphological sequences performed (T1, T2, FLAIR or DWI sequences).

**Figure 7:**
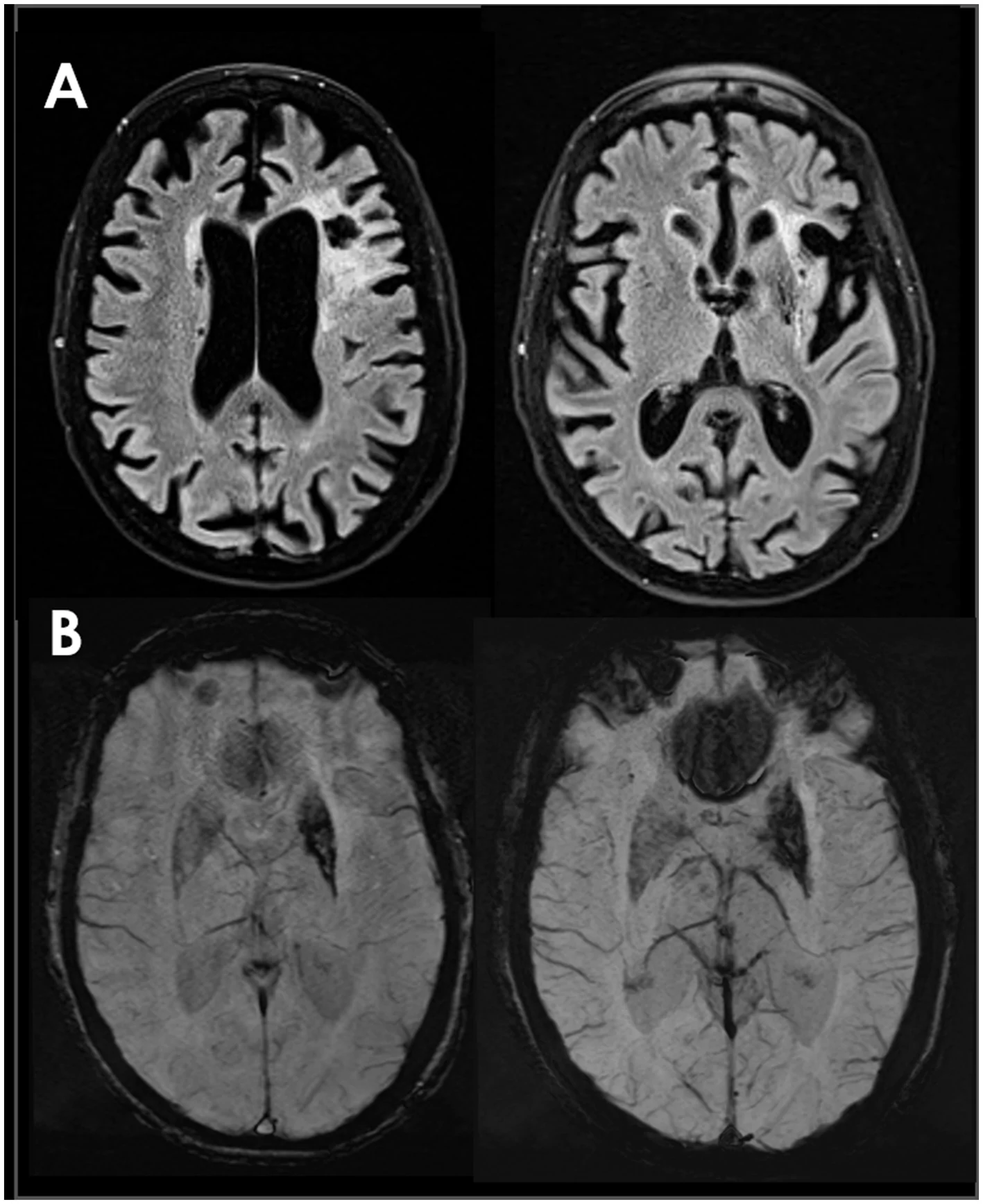
FLAIR **(A)** and SWI **(B)** sequences. Extensive area of postischemic encephalomalacia in left inferior frontal and insular gyri, with subcortical leukopathy and expansion of the ventricular horn. Associated asymmetric deposition of paramagnetic substances in the left lenticular nucleus **(B)**.

Crucially, longitudinal neuroimaging analysis in surgical patients with available pre-operative datasets confirmed that this deposition was absent prior to the operative intervention.

Additionally, our data indicate that this accumulation does not represent a transient radiological alteration following an acute event, such as surgery or an acute ischemic stroke. Once this deposition occurs, it remains constant or increases over time during follow-up (Figure 8).

**Figure 8:**
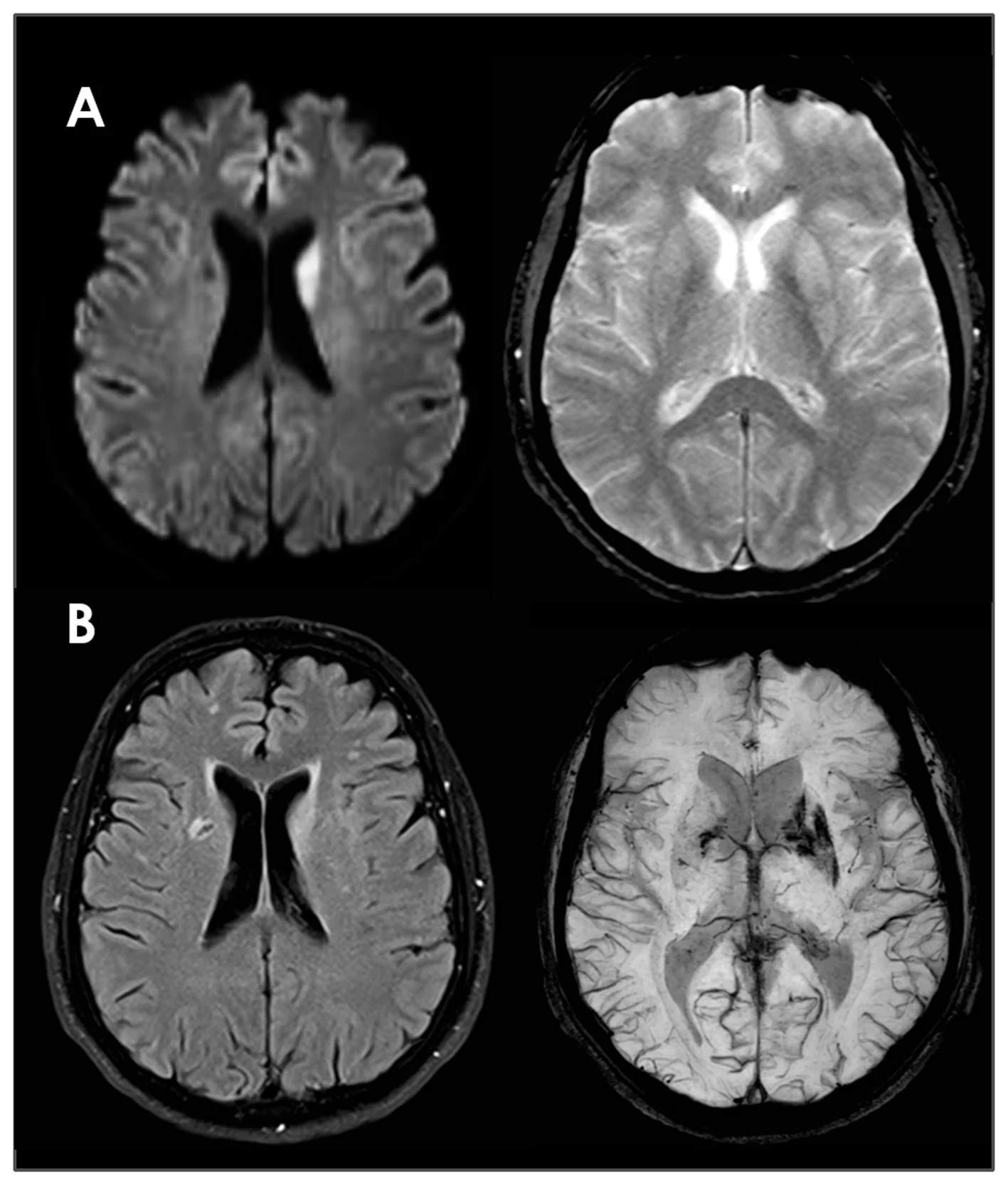
DWI and GRE **(A)**, FLAIR and SWI sequences 2 years later **(B)**. Acute ischemic lesion on the left caudate nucleus, without deposit of paramagnetic substances in the basal ganglia **(A)**. Gliosis and loss of caudate volume, with deposit of paramagnetic substances in the ipsilateral striatum nucleus **(B)**.

Finally, a notable case in our cohort involved a patient with Multiple Sclerosis presenting with a chronic active periventricular lesion (Figure 9), who demonstrated a marked, ipsilateral asymmetric deposition of paramagnetic substances within the basal ganglia.

**Figure 9:**
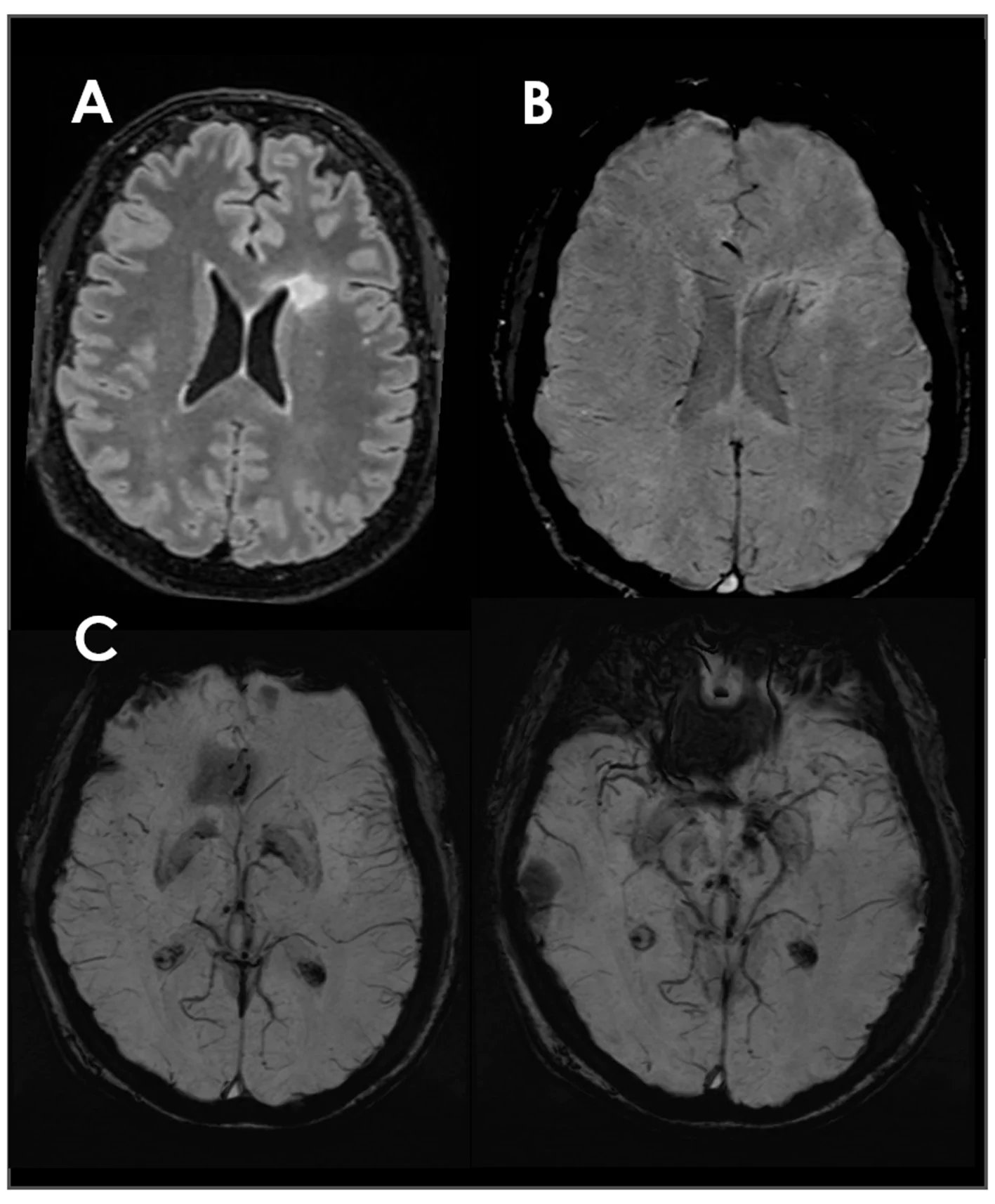
FLAIR **(A)**, and SWI **(B, C)** sequences. Frontal periventricular smoldering lesion with a ring of paramagnetic substances, and asymmetric deposition of paramagnetic substances in the ipsilateral left globus pallidus and SN.

## 4 Discussion

Degeneration of the basal ganglia and, consequently, their dysfunction, can lead to serious neurological conditions. The characteristic feature of basal ganglia injury is a movement disorder in which there is very little movement (hypokinesia), excessive movement (hyperkinesia), or a combination of both, depending on the location and extent of the affected structure (1, 2).

The presence of secondary degeneration in the SNpc means an irreversible loss of neuronal function because there is little evidence of axonal regeneration in the SNpc (14, 17). Consequently, the early *in vivo* identification of this phenomenon is highly valuable for predicting the long-term clinical trajectory of these patients.

Indeed, secondary neurodegeneration in the SNpc is a little-researched phenomenon Timely diagnosis at the early stage could aid in the design of neuroprotective assays and provide prognostic information on clinical outcomes (16).

After any damage, excitotoxic mechanisms and oxidative stress occur in the acute stage (5–12 weeks after the primary injury); that produces cytotoxic oedema and axonal fragmentation, followed by rupture of the myelin sheath (18, 19).

These early, distant microstructural alterations can be effectively monitored using diffusion-weighted imaging (DWI) sequences. A highly representative alteration within the subcortical nuclei consists of a transient diffusion restriction confined exclusively to the striatum following frontal lobe surgery or cortical ablation, reflecting an acute disruption of the frontostriatal projections (20). This diffusion restriction, which has been documented in the early phases of both Wallerian and transneuronal secondary degeneration, typically manifests within days post-injury, reaching its minimum Apparent Diffusion Coefficient (ADC) values at approximately 7 days before returning to baseline signal intensity around day 30 (21).

As the cascade progresses into the subacute stage, the loss of myelinated fibers accelerates, accompanied by local macrophage infiltration. Finally, during the chronic phase (>12 weeks post-injury), the degenerated axonal fibers are engulfed and replaced by fibrillary gliosis, culminating in an astrocytic response, localized tissue scarring, and terminal tract atrophy (22).

Iron accumulation in the brain can be measured *in vivo* via MR, with specific iron-sensitive sequences, such as Gradient Echo (GE) T2–weighted images, and susceptibility-weighted (SWI) images, and can be quantified by T2 mapping and Quantitative Susceptibility Mapping (QSM) (7, 18, 23, 24). This effect increases with the magnetic field and is therefore greater at 3T than at 1.5T.

Susceptibility-Weighted Imaging (SWI) sequence (23) may also show good correlation with the severity of clinical symptoms. The SWI sequence combines magnitude datasets—essential for anatomical evaluation—with filtered phase datasets that are uniquely sensitive to local variations in magnetic susceptibility (23). Tissues whose susceptibility properties diverge from the surrounding background, such as myelinated tracts or iron-rich proteins, induce local microenvironmental deviations in the magnetic field. This mechanism generates regional phase shifts that are extracted to optimize tissue contrast, enhancing the visualization of small deep brain structures and microvessels (23).

In the present cohort, an asymmetric deposition of paramagnetic substances was identified within the basal ganglia, systematically associated with an ipsilateral supratentorial, predominantly frontal, noxa (such as cerebral infarction, haematoma, tumor or demyelinating lesion). As demonstrated in our results, this deposition is not associated with other morphological alterations of these basal ganglia, such as atrophy, gliosis or necrosis on structural T1, T2, or FLAIR sequences; nor is it justified in the context of anterograde degeneration of this cortical-spinal pathway, although such Wallerian degeneration does exist in up to 30% of cases.

No significant differences were identified between the qualitative characteristics of the basal ganglia susceptibility changes and the primary etiology of the preceding pathology; instead, the manifestation appears to be more closely associated with the specific anatomical location and structural extent of the underlying condition.

Clinical translation could be highly relevant, since this asymmetrical deposition of paramagnetic substances is an early, subclinical or preclinical radiological finding, since up to 50% of these patients did not have associated symptoms.

Furthermore, in patients who underwent neurosurgery and had pre-operative neuroimaging available, longitudinal analysis confirmed that this specific asymmetric deposition did not exist prior to the surgical intervention.

Another fact to point out is that this deposition does not occur in the context of a transient alteration after an acute event such as surgery or acute ischemic injury. As documented in the presented results, this manifestation represents a progressive process that develops over extended timelines once the primary neuronal damage has occurred. Once this deposition has occurred it remains constant or increases over time, independently of the etiology, the size, or the location of the preexistent lesion. This temporal evolution suggests an ongoing, active metabolic shift within the subcortical nuclei rather than an acute inflammatory artifact (25).

Advanced chronological age was found to be a decisive factor, as the presence of this asymmetric deposition appeared significantly more pronounced in older patients within the cohort. This correlation aligns with previous literature establishing age as a critical accelerator of neurodegeneration linked to iron dyshomeostasis (26–30).

Upon analysis of these susceptibility changes, the condition appears significantly more prevalent within the ipsilateral globus pallidus and substantia nigra, whereas it occurs less frequently within the neostriatal nuclei. This finding is significant and supports the hypothesis of transsynaptic degeneration, since the globus pallidus and the SN form the output pathway of the information transmitted through the basal ganglia to the thalamus, for the modulation of the motor circuit (2, 5, 6). Interestingly, abnormal deposition of paramagnetic substances within the thalamus of the involved cerebral hemisphere was not identified in any of the cases; however, a unique instance was documented in which primary thalamic involvement retrogradely affected the ipsilateral striatum.

Also noteworthy is the case of a patient with Multiple Sclerosis, with a chronic active periventricular lesion, whose ipsilateral asymmetric deposition of paramagnetic substances in the basal ganglia would support statements of the latest articles, which associate these smoldering lesions with demyelination and neurodegeneration, with a worse clinical prognosis. Pathophysiologically, this specific case highlights how deafferentation and localized neuroinflammation can alter regional iron homeostasis (8, 12).

While the preliminary evidence presented here is derived from a retrospective cohort of 35 cases characterized by etiological heterogeneity, this study serves as an important proof-of-concept. The data support the hypothesis that an asymmetric deposition of paramagnetic substances within the basal ganglia occurs during the chronic phases of transsynaptic neurodegeneration secondary to distant supratentorial injury, demonstrating a high predilection for frontostriatal pathways. This structural and metabolic shift can be identified early on MRI via the SWI sequence (23), long before conventional structural alterations associated with advanced neurodegeneration—such as tissue gliosis or macroscopic atrophy—become visible. Consequently, this radiological sign may broaden the current understanding of the complex functional connectivity of cortico-basal networks and the metabolic dynamics of transsynaptic neurodegeneration. Furthermore, it holds potential for future development as a non-invasive surrogate biomarker providing early prognostic utility for the longitudinal management and monitoring of patients suffering from focal supratentorial injuries.

Finally, this transsynaptic theoretical framework may offer a novel pathophysiological explanation for a historically controversial phenomenon: the delayed deposition of paramagnetic substances within the contralateral globus pallidus following a primary hemorrhage or hemorrhagic infarction in the opposite hemisphere. This contralateral alteration has traditionally been attributed to a passive “blood mobilization” migrating through the Gratiolet channel. Based on the observations from this cohort, it is hypothesized that such delayed contralateral changes may instead represent a secondary transsynaptic degeneration accompanied by localized metabolic iron accumulation, driven by the interruption of interhemispheric or commissural regulatory loops.

## 5 Limitations

Several inherent limitations must be acknowledged within the context of the present study, chief among which are its retrospective design, single-center execution, relatively small sample size, and constrained longitudinal follow-up period, alongside the pronounced clinical and etiological heterogeneity of the primary supratentorial lesions. Multi-center studies encompassing extended longitudinal observation windows remain necessary to further corroborate and consolidate these radiological findings within routine clinical practice.

Given the retrospective nature of case recruitment based on the presence of the imaging sign, the true prevalence, sensitivity, and specificity of this finding cannot be mathematically determined from the current dataset. The absence of a matched control group (composed of either healthy individuals or patients presenting with equivalent frontal injuries but lacking deep gray matter alterations) limits the ability to statistically define whether this interhemispheric asymmetry systematically exceeds natural, physiological variation.

Methodologically, this study relies mainly on visual assessment on the qualitative of SWI findings, without QSM or susceptibility asymmetry measurements. While SWI is highly sensitive to paramagnetic substances (23), emerging quantitative susceptibility mapping (QSM) sequences (7, 31) will be essential in future prospective studies to precisely quantify iron volume and assess long-term longitudinal changes.

Another interesting factor would be to be able to confirm this hypothesis with anatomopathological data, which would definitively validate the microstructural substrate of this transsynaptic iron deposition. Nevertheless, the strength of our diverse sample lies in demonstrating that this transsynaptic iron deposition is independent of the primary etiology, highlighting a universal mechanism of network disruption.

## 6 Conclusions

The pathophysiology of the neural networks between the cerebral cortex and the basal ganglia remains highly intricate, and the integration of advanced MR sequences could help to complete the existing knowledge.

This article describes a novel radiological finding consisting of an asymmetric deposition of paramagnetic substances in the ipsilateral basal ganglia associated to a mainly frontal supratentorial noxa. This hitherto undescribed phenomenon could provide new insights into frontostriatal network pathophysiology and could be explained as an early biomarker in the context of transsynaptic degeneration, with potential prognostic clinical implications, as well as to expand the existing knowledge of the physiology of these cortico-basal circuits.

## Data Availability

The raw data supporting the conclusions of this article will be made available by the authors, without undue reservation.

## References

[1] Purves D Augustine GJ Fitzpatrick D Hall WC LaMantia AS Mooney RD ., *Neuroscience*, 6th Edn. New York, NY: Oxford University Press (2018).

[2] Lanciego JL Luquin N Obeso JA. Functional neuroanatomy of the basal ganglia. Cold Spring Harb Perspect Med. (2012) 2:a009621. doi: 10.1101/cshperspect.a009621 23071379 PMC3543080

[3] Haber SN. The primate basal ganglia: parallel and integrative networks. J Chem Neuroanat. (2003) 26:317–30. doi: 10.1016/j.jchemneu.2003.10.003 14729134

[4] Zhou FM Lee CR. Intrinsic and integrative properties of substantia nigra pars reticulata neurons. Neuroscience. (2011) 198:69–94. doi: 10.1016/j.neuroscience.2011.07.061 21839148 PMC3221915

[5] Alexander GE DeLong MR Strick PL. Parallel organization of functionally segregated circuits linking basal ganglia and cortex. Annu Rev Neurosci. (1986) 9:357–81. doi: 10.1146/annurev.ne.09.030186.002041 3085570

[6] Nambu A Tokuno H Takada M. Functional significance of the hyperdirect pathway: integrated signal processing in the basal ganglia. Neurosci Res. (2002) 43:111–7. doi: 10.1016/S0168-0102(02)00027-5 12067746

[7] Langkammer C Bredies K Poser BA Barth M Reishofer G Fan AP . Fast quantitative susceptibility mapping using 3D EPI and total generalized variation. Neuroimage. (2015) 111:622–30. doi: 10.1016/j.neuroimage.2015.02.041 25731991

[8] Ward RJ Zucca FA Duyn JH Crichton RR Zecca L. The role of iron in brain ageing and neurodegenerative disorders. Lancet Neurol. (2014) 13:1045–60. doi: 10.1016/S1474-4422(14)70117-6 25231526 PMC5672917

[9] Acosta-Cabronero J Betts MJ Cardenas-Blanco A Yang S Nestor PJ. *In vivo* MRI mapping of brain iron deposition across the adult lifespan. J Neurosci. (2016) 36:364–74. doi: 10.1523/JNEUROSCI.1907-15.2016 26758829 PMC4710766

[10] Steiger TK Weiskopf N Bunzeck N. Iron level and myelin content in the ventral striatum predict memory performance in the aging brain. J Neurosci. (2016) 36:3552–8. doi: 10.1523/JNEUROSCI.3617-15.2016 27013683 PMC4804012

[11] Xu J Jia Z Knutson MD Leeuwenburgh C. Impaired iron status in aging research. Int J Mol Sci. (2012) 13:2368–86. doi: 10.3390/ijms13022368 22408459 PMC3292028

[12] Benarroch EE. Brain iron homeostasis and neurodegenerative disease. Neurology. (2009) 72:1436–40. doi: 10.1212/WNL.0b013e3181a26b30 19380704

[13] Onen MR Moore ML Ginat DT. MR imaging of CNS tractopathy: wallerian and transneuronal degeneration. AJNR Am J Neuroradiol. (1998) 19:813–8. doi: 10.2214/ajr.171.3.9725322 9725322

[14] Zedde M Grisendi I Assenza F Napoli M Moratti C Valzania F . Secondary neurodegeneration of ipsilateral substantia nigra in acute ischemic stroke. Neurol Sci. (2023) 44:4099–102. doi: 10.1007/s10072-023-06972-w 37526798

[15] Ogawa T Yoshida Y Okudera T Noguchi K Kado H Uemura K. Secondary thalamic degeneration after cerebral infarction in the middle cerebral artery distribution: evaluation with MR imaging. Radiology. (1997) 204:255–62. doi: 10.1148/radiology.204.1.9205256 9205256

[16] Block F Dihne M Loos M. Inflammation in areas of remote changes following focal brain lesion. Prog Neurobiol. (2005) 75:342–65. doi: 10.1016/j.pneurobio.2005.03.004 15925027

[17] Zhao F Kuroiwa T Miyasaka N Nagaoka T Nakane M Tamura A . Characteristic changes in T(2)-value, apparent diffusion coefficient, and ultrastructure of substantia nigra evolving exofocal postischemic neuronal death in rats. Brain Res. (2001) 895:238–44. doi: 10.1016/S0006-8993(00)03281-9 11259783

[18] Gutteridge JM Westermarck T Santavuori P. Iron and oxygen radicals in tissue damage: implications for the neuronal ceroid lipofuscinoses. Acta Neurol Scand. (1983) 68:365–70. doi: 10.1111/j.1600-0404.1983.tb04845.x 6666544

[19] Singh A Kukreti R Saso L Kukreti S. Oxidative stress: a key modulator in neurodegenerative diseases. Molecules. (2019) 24:1583. doi: 10.3390/molecules24081583 31013638 PMC6514564

[20] Kamiya K Sato N Nakata Y Ito K Kimura Y Ota M . Postoperative transient reduced diffusion in the ipsilateral striatum and thalamus. AJNR Am J Neuroradiol. (2013) 34:524–32. doi: 10.3174/ajnr.A3242 22899787 PMC7964900

[21] Montoya-Filardi A Garc-Junco Albacete M Ortol1 Fortes P Carreres Polo J. Carretera perdida: imagen de la degeneración neuronal secundaria. Radiolog. (2022) 64:145–55. doi: 10.1016/j.rx.2022.01.008 35504680

[22] Chen YJ Nabavizadeh SA Vossough A Kumar S Loevner LA Mohan S. Wallerian degeneration beyond the corticospinal tracts: conventional and advanced MRI findings. J Neuroimaging. (2017) 27:272–80. doi: 10.1111/jon.12404 28072502

[23] Haacke EM Reichenbach JR. Susceptibility Weighted Imaging in MRI: Basic Concepts and Clinical Applications. Hoboken, NJ: Wiley–Blackwell (2011). doi: 10.1002/9780470905203

[24] Ghadery C Pirpamer L Hofer E Langkammer C Petrovic K Loitfelder M . R2^*^ mapping for brain iron: associations with cognition in normal aging. Neurobiol Aging. (2015) 36:925–32. doi: 10.1016/j.neurobiolaging.2014.09.013 25443291

[25] Loos M Dihne M Block F. Tumor necrosis factor-alpha expression in areas of remote degeneration following middle cerebral artery occlusion of the rat. Neuroscience. (2003) 122:373–80. doi: 10.1016/S0306-4522(03)00498-6 14614903

[26] Bartzokis G Tishler TA Lu PH Villablanca P Altshuler LL Carter M . Brain ferritin iron may influence age- and gender-related risks of neurodegeneration. Neurobiol Aging. (2007) 28:414–23. doi: 10.1016/j.neurobiolaging.2006.02.005 16563566

[27] Xu X Wang Q Zhang M. Age, gender, and hemispheric differences in iron deposition in the human brain: an *in vivo* MRI study. Neuroimage. (2008) 40:35–42. doi: 10.1016/j.neuroimage.2007.11.017 18180169

[28] Hallgren B Sourander P. The effect of age on the non-haemin iron in the human brain. J Neurochem. (1958) 3:41–51. doi: 10.1111/j.1471-4159.1958.tb12607.x 13611557

[29] Daugherty A Raz N. Age-related differences in iron content of subcortical nuclei observed *in vivo*: a meta-analysis. Neuroimage. (2013) 70:113–21. doi: 10.1016/j.neuroimage.2012.12.040 23277110 PMC3580001

[30] Ramos P Santos A Pinto NR Mendes R Magalhães T Almeida A. Iron levels in the human brain: a post-mortem study of anatomical region differences and age-related changes. J Trace Elem Med Biol. (2014) 28:13–7. doi: 10.1016/j.jtemb.2013.08.001 24075790

[31] Zhang Y Wei H Cronin MJ He N Yan F Liu C. Longitudinal atlas for normative human brain development and aging over the lifespan using quantitative susceptibility mapping. Neuroimage. (2018) 171:176–89. doi: 10.1016/j.neuroimage.2018.01.008 29325780 PMC5857468

